# Distress Management in Patients With Head and Neck Cancer Before Start of Palliative Chemotherapy: A Practical Approach

**DOI:** 10.1200/JGO.17.00044

**Published:** 2018-01-30

**Authors:** Vijay Patil, Vanita Noronha, Amit Joshi, Jayita Deodhar, Savita Goswami, Santam Chakraborty, Anant Ramaswamy, Sachin Dhumal, Chandrakanth M.V., Ashay Karpe, Nikhil Pande, Vikas Talreja, Arun Chandrasekharan, Siddharth Turkar, Kumar Prabhash

**Affiliations:** **Vijay Patil**, **Vanita Noronha**, **Amit Joshi**, **Jayita Deodhar**, **Savita Goswami**, **Santam Chakraborty**, **Anant Ramaswamy**, **Sachin Dhumal**, **M.V. Chandrakanth**, **Ashay Karpe**, **Nikhil Pande**, **Vikas Talreja**, **Arun Chandrasekharan**, **Siddharth Turkar**, and **Kumar Prabhash**, Tata Memorial Centre, Mumbai, India.

## Abstract

**Purpose:**

This study reports the incidence of distress, the factors associated with distress, and a practical strategy to resolve distress in patients with head and neck cancer who are starting palliative chemotherapy.

**Methods:**

Adult patients with head and neck cancer planned for palliative chemotherapy underwent distress screening before the start of treatment as part of this single-arm prospective study. Patients who had a distress score > 3 on the National Comprehensive Cancer Network (NCCN) distress thermometer were counseled initially by the clinician. Those who continued to have high distress after the clinician-led counseling were referred to a clinical psychologist and were started on palliative chemotherapy. After counseling, distress was measured again. The relation between baseline distress and compliance was tested using Fisher's exact test.

**Results:**

Two hundred patients were enrolled, and the number of patients with high distress was 89 (44.5% [95% CI, 37.8% to 51.4%]). The number of patients who had a decrease in distress after clinician-led counseling (n = 88) was 52 (59.1% [95% CI, 48.6% to 68.8%]) and after psychologist-led counseling (n = 32) was 24 (75.0% [95% CI, 57.6% to 72.2%]; *P* = .136). Compliance rates did not differ between the patients with or without a high level of distress at baseline (74.2% *v* 77.4%, *P* = .620).

**Conclusion:**

The incidence of baseline distress is high in patients awaiting the start of palliative chemotherapy. It can be resolved in a substantial number of patients using the strategy of clinician-led counseling, with additional referral to a clinical psychologist as required. Patients with a greater number of emotional problems usually require psychologist-led counseling.

## INTRODUCTION

Distress is the sixth vital sign.^1^ It must be monitored at every important decision-making point in patients with cancer.^2^ Distress management is a necessity because patients with high distress are frequently noncompliant with treatment protocols and follow-up.^3,4^ In addition, high distress is associated with a poor quality of life in patients with cancer.^5^ The authors of this study had previously conducted a feasibility study on the use of the National Comprehensive Cancer Network (NCCN) distress thermometer (DT) for distress screening in south India. In that study, 80% of patients undergoing palliative treatment had a high distress score. Furthermore, > 80% of patients with high distress scores had emotional problems, nearly 50% had practical problems, and all patients (100%) had physical problems.^6^ These data stressed the need to consider distress screening in routine practice. Patients with head and neck cancer who are undergoing palliative chemotherapy frequently have physical symptoms, disfigurement, and disablement and are socially isolated.^7-11^ Hence, distress screening in such patients is of prime importance.^12^

This study was designed to capture the expectations and preferences of patients with head and neck cancer warranting palliative chemotherapy and to identify the incidence of distress and the factors associated with it in these patients. The expectations and preferences of these patients have already been published elsewhere.^13^ This study concentrates on distress-related key secondary end points. Routine distress screening may not be feasible in developing nations because of limited manpower resources.^6^ A clinical psychologist may not be available in each cancer site’s outpatient department. Furthermore, in centers with a high patient load, it is not feasible for each patient with high distress to be counseled by a psychologist in routine practice.^14^ Hence, a practical strategy was considered and tested in this study. In this strategy, patients with high distress were counseled initially by the treating physician and were referred to a clinical psychologist only if the distress was high after this clinician-led counseling. In this post hoc analysis, we studied the efficacy of this strategy in relieving distress.

## METHODS

### Eligibility Criteria

Adult patients with head and neck cancer who are planned for treatment with palliative chemotherapy were enrolled in this study. Treatment decisions were made for all patients after a multidisciplinary joint clinical discussion. Details of the inclusion and exclusion criteria for this study are published elsewhere.^13^

### Study Design

This was a prospective, single-arm, observational study conducted in the Department of Medical Oncology of Tata Memorial Centre. Before starting palliative chemotherapy, patients underwent protocol-defined structured counseling that included details of diagnosis, stage of disease, prognosis, benefits and risks of chemotherapy, cost of chemotherapy, precautions to be taken during chemotherapy, and details of financial assistance schemes. The protocol-defined structured counseling proforma is provided in the Appendix (online only). After counseling, patients were administered the NCCN DT by the physicians.

The NCCN DT is a validated tool for distress screening that is composed of a graphical representation of a thermometer marked from 1 to 10, on which patients mark their perceived level of distress. In addition, a problem list is provided, in which 37 problems in six domains are listed.^15^ Patients with a distress score of ≥ 4 were considered to have high distress and were asked to fill in the problem list as well.^2^

Patients who had a distress score of < 4 on the DT were started on palliative chemotherapy and were followed up at 2-month intervals. Patients who had a distress level of ≥ 4 on the DT were counseled by the one of the clinicians. Counseling focused on tackling the distress according to the problems identified by the patient in the problem list. Examples of counseling points included symptomatic treatment and its benefit for patients with concerns regarding physical problems, financial support schemes and its access when financial problems were identified, and assurance of an early start of treatment. The counseling was conducted in the outpatient department clinic itself on the same day. The average time spent in counseling varied with the number of problems identified on the problem list, but it took an average of 10 minutes.

The NCCN DT was readministered immediately after this counseling, and patients who still had a distress level of ≥ 4 were referred to a psychologist. Patients were counseled by the psychologist, after which distress was measured again using the DT. An appointment with the psychologist was placed within 48 hours. The palliative chemotherapy was started after the psychologist counseling. Patients were followed up at 2-month intervals. The follow-up continued as long as the patients were alive.

### Study Oversight

This investigator-initiated study was approved by the institutional ethics committee of Tata Memorial Centre. The study protocol was registered with the Clinical Trial Registry of India (CTRI/2015/11/006392). All patients gave their written informed consent before enrollment in the study, and the study was conducted in accordance with good clinical practice guidelines and the Declaration of Helsinki.

### Statistical Analysis

The statistical analysis was performed using SPSS (SPSS, Chicago, IL) and R studio. Descriptive statistics in the form of frequencies were calculated to describe the distress at baseline. The efficacy of clinician and psychologist-led counseling was expressed in percentages with their respective 95% CIs. Binary logistic regression analysis was performed to determine the factors associated with high baseline distress. The factors studied were age (≥ 70 years or < 70 years), sex (male or female), socioeconomic status (above the poverty line or below the poverty line), and residential address (Mumbai, rest of Maharashtra, or rest of India).^12,16-18^ Income of < 1.9 USD per day was considered to be below the poverty line.

A binary logistic regression analysis was also performed to identify patients in whom distress was resolved with clinician-led counseling. The factors tested were the number of problems in each domain, considered as a continuous variable per domain. The domains consisted of practical problems (maximum count: 5), emotional problems (maximum count: 6), family problems (maximum count: 4), spiritual problems (maximum count: 1), and physical problems (maximum count: 21).

We considered patients as noncompliant if any one of the following events had occurred: (1) the patient was lost to follow-up, defined as missing any visit between baseline and the 6-month visit; a delay in attending a scheduled visit by > 15 days without prior information was also considered to be lost to follow-up; (2) the patient received treatment at an outside center whose protocol diverged from that at our center; or (3) the patient attended for regular follow-up but was not taking chemotherapy medications regularly.

Patients who complied with the study protocol until 6 months after the start of therapy were considered to be compliant. The association between baseline distress and compliance was tested using Fisher's exact test. A two-sided *P* value of ≤ .05 was taken as significant.

## RESULTS

### Baseline Distress, Problem List, and Factors Affecting Distress

Two hundred patients were enrolled in this study between December 2015 and April 2016, and data regarding baseline distress were present in all patients. The median distress score was 3 (range, 0 to 10). Eighty-nine patients (44.5% [95% CI, 37.8% to 51.4%]) were found to have high distress (ie, a distress score of ≥ 4 on the DT). Table 1 provides the details of the problems listed by these 89 patients. The major physical and practical problems experienced were related to pain, change in appearance, and concerns regarding finance or insurance. These were present in nearly two thirds of the patient population with a high distress score. Table 2 provides the details of the baseline patient-related factors that we postulated may affect distress. None of the factors tested were found to be associated with high distress. None of the patients had problems confined to only a singular domain on the problem list.

**Table 1:**
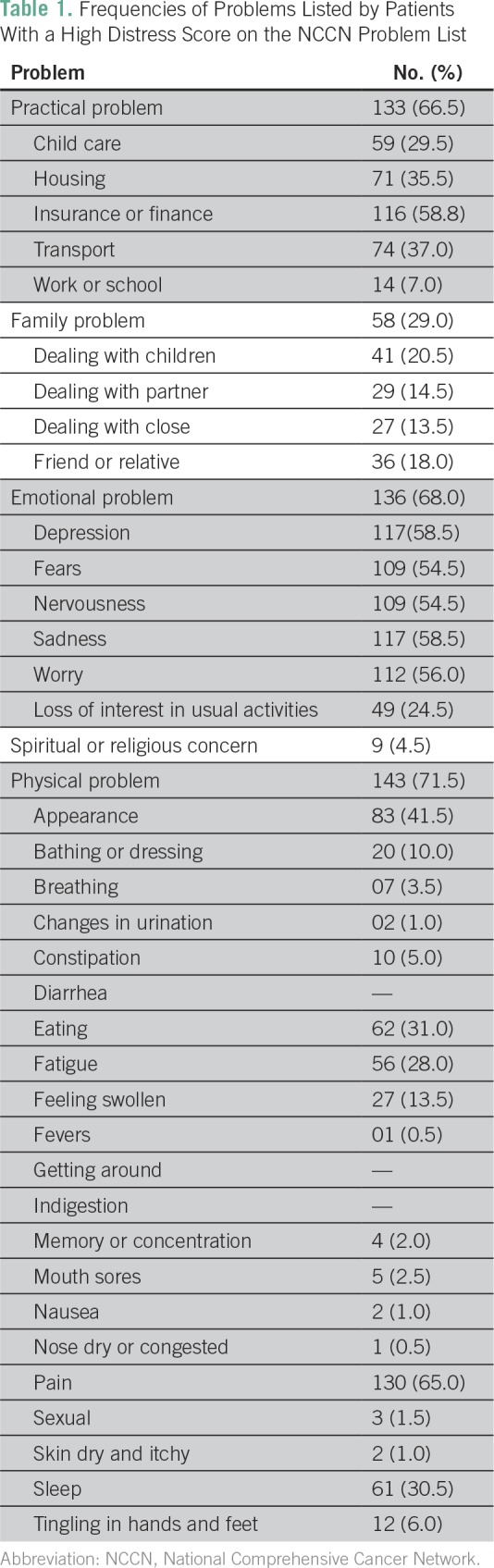
Frequencies of Problems Listed by Patients With a High Distress Score on the NCCN Problem List

**Table 2:**
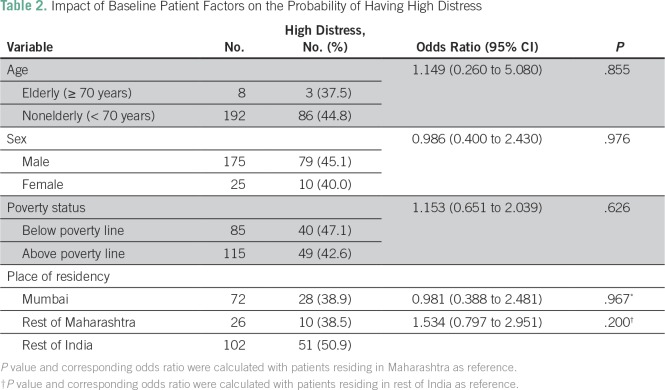
Impact of Baseline Patient Factors on the Probability of Having High Distress

### Efficacy of Clinician-Led and Psychologist-Led Counseling

Among the 89 patients who had high distress, 88 patients (98.9%) underwent clinician-led counseling. Of these 88 patients, 52 (59.1% [95% CI, 48.6% to 68.8%]) had a reduction in their distress score to < 4 after the initial counseling by the clinician. One patient refused clinician-led counseling and hence was started directly on palliative chemotherapy. Patients with a greater number of emotional problems had a persistence of high distress in clinical counseling on binary logistic regression analysis. Details are listed in Table 3. Thirty-six patients were referred for psychological counseling in view of their high distress score. Of these, four patients refused counseling with a clinical psychologist. The number of patients in whom the distress decreased (to a distress score of < 4) after counseling by a trained clinical psychologist was 24 (75.0% [95% CI, 57.6% to 72.2%]).

**Table 3:**
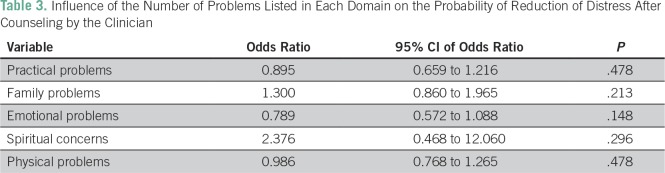
Influence of the Number of Problems Listed in Each Domain on the Probability of Reduction of Distress After Counseling by the Clinician

### Impact of Distress on Compliance

Of the 200 patients enrolled in the study, 48 patients (24.0%) were noncompliant. Among these, 35 patients (17.5%) were lost to follow up, four patients (2.0%) changed their protocol-defined treatment, and nine patients (4.5%) were noncompliant in taking the prescribed oral chemotherapy. Patients with a high baseline distress score (score ≥ 4) had the same compliance rate as patients with a low distress score (score of < 4; 74.2% *v* 77.45, *P* = .62). Similarly, we observed no difference in the compliance rates between patients in whom distress was ameliorated after clinician-led counseling and those in whom high distress (score ≥ 4) persisted (75% *v* 72%, *P* = .809; Table 4). A total of 62.5% of patients with persistent distress after the second round of counseling, led by the clinical psychologist, were compliant with additional therapy. In contrast, 83.3% of the patients in whom distress decreased after the second round of counseling were found to be compliant with additional therapy and follow-up. This difference was not statistically significant (*P* = .327).

**Table 4:**
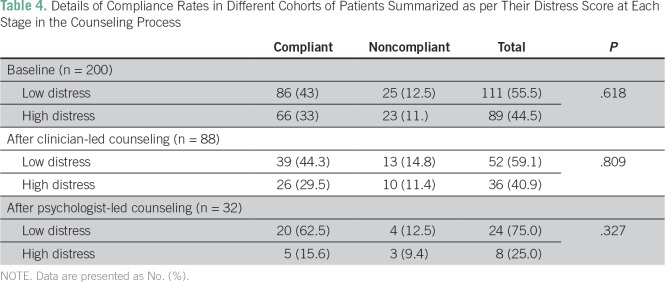
Details of Compliance Rates in Different Cohorts of Patients Summarized as per Their Distress Score at Each Stage in the Counseling Process

### Distress and Overall Survival

The median overall survival in patients with high baseline distress (score of ≥ 4) was 199 days (95% CI, 156.6 to 241.4 days), whereas the median overall survival in patients with low distress was 193 days (95% CI, 177.7 to 208.3 days; *P =* .880). However, the cohort of patients who had a high distress score after psychologist-led counseling had a numerically inferior median overall survival (145 days *v* 198 days, *P =* .331).

## DISCUSSION

Head and neck cancers are the most common cancers in India, with a 5-year prevalence of 1,45,087 cases.^19^ These cancers are associated with high rates of distress.^7,9,10,12,20,21^ The incidence of suicide, depression, and anxiety is also high in these cancers.^5,22-24^ Among patients with head and neck cancer, those awaiting the start of palliative chemotherapy are expected to have the greatest degree of distress. It is often felt that detailed counseling about the disease status and its prognosis, the risks and benefits of palliative chemotherapy, and its cost and duration may further contribute toward this distress, especially in patients who lack financial, social, and practical resources (stay, transport, and so forth).^25^

To the best of our knowledge, before this study, distress had never been studied systematically in patients with head and neck cancer who are undergoing palliative chemotherapy. Our data highlight the importance of this distress, because we have shown that nearly 40% of patients have high distress after counseling. However, this is substantially less than the prevalence of distress noted in similar patient groups, which may be indicative of the fact that detailed counseling regarding the treatment and prognosis may not result in high distress. Although we cannot definitely make a comment on this because baseline distress before the physician-led counseling was not captured, several patients indicated to the authors that they felt significant relief after the initial counseling regarding disease status, overall prognosis, and the benefit of the palliative chemotherapy offered.

Counseling by a psychologist is recommended in actionable distress.^2^ However, in countries like ours and even globally, there is a shortage of clinical psychologists.^14^ The strategy evaluated in this study for distress counseling was structured keeping in mind that limitation. The efficacy of clinician-led counseling was reassuring, with nearly two thirds of patients having a reduction in their distress.

As expected, counseling by a clinical psychologist showed high efficacy, with distress being relieved in nearly two thirds of patients. The lead author had performed a distress screening study in south India in which 80% of patients treated with palliative treatment were found to have high distress. This figure led us to assume that a greater number of patients would have distress when the study was planned and that the sample size of 200 patients would provide sufficient power for such analysis. However, this was not the case in this study; only 89 patients (44.5%) had high distress, and only 32 patients required psychologist-led counseling after the initial clinician-led counseling. As a result, although a higher proportion of patients did derive benefit from psychologist-led counseling, the difference was not statistically significant. This lack of statistical significance should not detract from the fact that every patient with high distress should ideally be counseled by a psychologist. We had hypothesized that patients with high distress because of emotional problems may not experience relief after clinician-led counseling. This was confirmed in this study; each increase in the number of emotional problems was associated with 1.26 times increased odds of having persistent high distress, although this finding was not statistically significant because of low numbers.

The compliance of patients in relation to their distress has been studied as well. Although this was a post hoc analysis, we adopted a rigorous definition of compliance. Because high distress is known to be associated with noncompliance with treatment protocols,^3,26^ similar compliance rates in patients with high and low baseline distress are an indicator of the efficacy of the proposed strategy in the resolution of distress. Clinical outcomes have not been studied because we await additional follow-up.

We also attempted to check for the factors associated with distress in this study. These factors were prespecified and were selected on the basis of a literature review conducted at the time of the drafting of the protocol.^12,16-18^ These protocol-specified factors were supposed to help us in triaging patients for implementation of measures directed toward relieving distress. However, as this study shows, baseline patient-related factors were not helpful in identifying a population of patients with distress. This, together with the multidomain list of problems identified by patients with high distress, attests to the fact that distress is a multifactorial phenomenon and is not usually explained or predicted by a single factor that is based on predetermined objective sociodemographic criteria.

This study stresses the need for head and neck physicians to spend time discussing ancillary concerns with patients, because these concerns contribute significantly toward patient distress. Reassuringly, in approximately 60% of patients, physician-led discussion can relieve distress. Thus, in resource-constrained settings, an initial round of clinician-led counseling may be adopted, followed by a triage system in which patients with multiple emotional problems are referred directly to a trained clinical psychologist.

This study is not without limitations. It is a single-center prospective study. The analysis for identifying the factors associated with the efficacy of clinician-led counseling was a post hoc analysis. However, the factors used in this analysis were collected prospectively and hence are unlikely to have influenced the study findings.

A substantial proportion of patients with head and neck cancer have high distress after initial counseling, and distress screening is necessary at this stage. Perceived distress can also be resolved in a substantial proportion of patients by problem-directed clinician-led counseling, followed by counseling by a trained clinical psychologist if high distress persists. However, patients who list a greater number of emotional problems usually require psychologist-led counseling upfront.

## Appendix

### Counselling

1. Stage of disease
2. Incurable nature
3. Prognosis: Median OS without chemotherapy 4-6 months 1 year survival below 10%, Median OS with chemotherapy 6-8 months, 1 year survival 10-20%, Median OS with Cetuximab based chemotherapy 8-10 months, 1 year survival 20-30%.
4. Relief in symptoms: Pain relief seen in nearly 60-70% of patients. The swelling would decrease subjectively in 50-60% of patients. The foul smell would decrease.
5. Side effects of chemotherapy
  - Cetuximab based (EXTREME study)
    - Any grade side effects: Cumulative percentage 80-100% (Common adverse events: anorexia, myalgia, allergic reactions, rash, diarrhea, fatigue, neuropathy)
    - Grade 3-4 side effects: Cumulative percentage 35-40% (Serious common adverse events: febrile neutropenia, grade 3-4 diarrhea, grade 3-4 rash, grade 3 fatigue, electrolyte imbalances, grade 3-4 neuropathy)
    - Death due to side effects: 4-5%
  - Metronomic chemotherapy (TMH study)
    - Any grade side effects: Cumulative percentage 60-80% (Common adverse events: anemia, fatigue and mucositis)
    - Grade 3-4 side effects: Cumulative percentage 15-20% (Serious common adverse events: febrile neutropenia, grade 3-4 mucositis, grade 3 fatigue, grade 3 fatigue, pneumonia)
    - Death due to side effects: <1%
  - Paclitaxel and carboplatin (Gibson et al, JCO 2005)
    - Any grade side effects: Cumulative percentage 80-100% (Common adverse events: anorexia, myalgia, allergic reactions, vomiting , diarrhea, fatigue, neuropathy)
    - Grade 3-4 side effects: Cumulative percentage 35-40% (Serious common adverse events: febrile neutropenia, grade 3-4 diarrhea, grade 3-4 mucositis, grade 3 fatigue, electrolyte imbalances, grade 3-4 neuropathy)
    - Death due to side effects: 4-5%
6. Routes of administration
7. Duration of treatment: till intolerable side effects or progression of disease
8. Cost as discussed above
  - Cetuximab based: 10 lakhs Rs/6 months of treatment
  - Metronomic: 5000 Rs/6 months of treatment
  - Paclitaxel platinum: 2 lakh rs/6 months of treatment
9. Inform regarding financial schemes and social worker support. Financial support decision has to be decided only after discussion with consultant.
10. Social support requirement
  - Need to stay in Mumbai/nearby place approachable to the hospital within 2-3 hours. If not available to counsel for taking chemotherapy at local place
  - Family support
11. Warning symptoms
  - If any of the following occurs the patients should report to TMH if taking chemotherapy with us or with the treating physician if opts for treatment outside
    - Fever (temperature 100 degree F or more)
    - More than 3 Vomitings or loose motions
    - Oral ulcers making it difficult for taking solid foods
    - Extreme fatigue or dizziness
    - Any issues which the patient feels is serious
  - If for any reason they are unable to come to hospital then they should report to local MD Physician for care
12. Advice regarding diet.
  - No outside eatables.
  - Preferably canned packed juices.
  - No fresh salads.
  - Anorexia would happen hence to take small regular diets.
